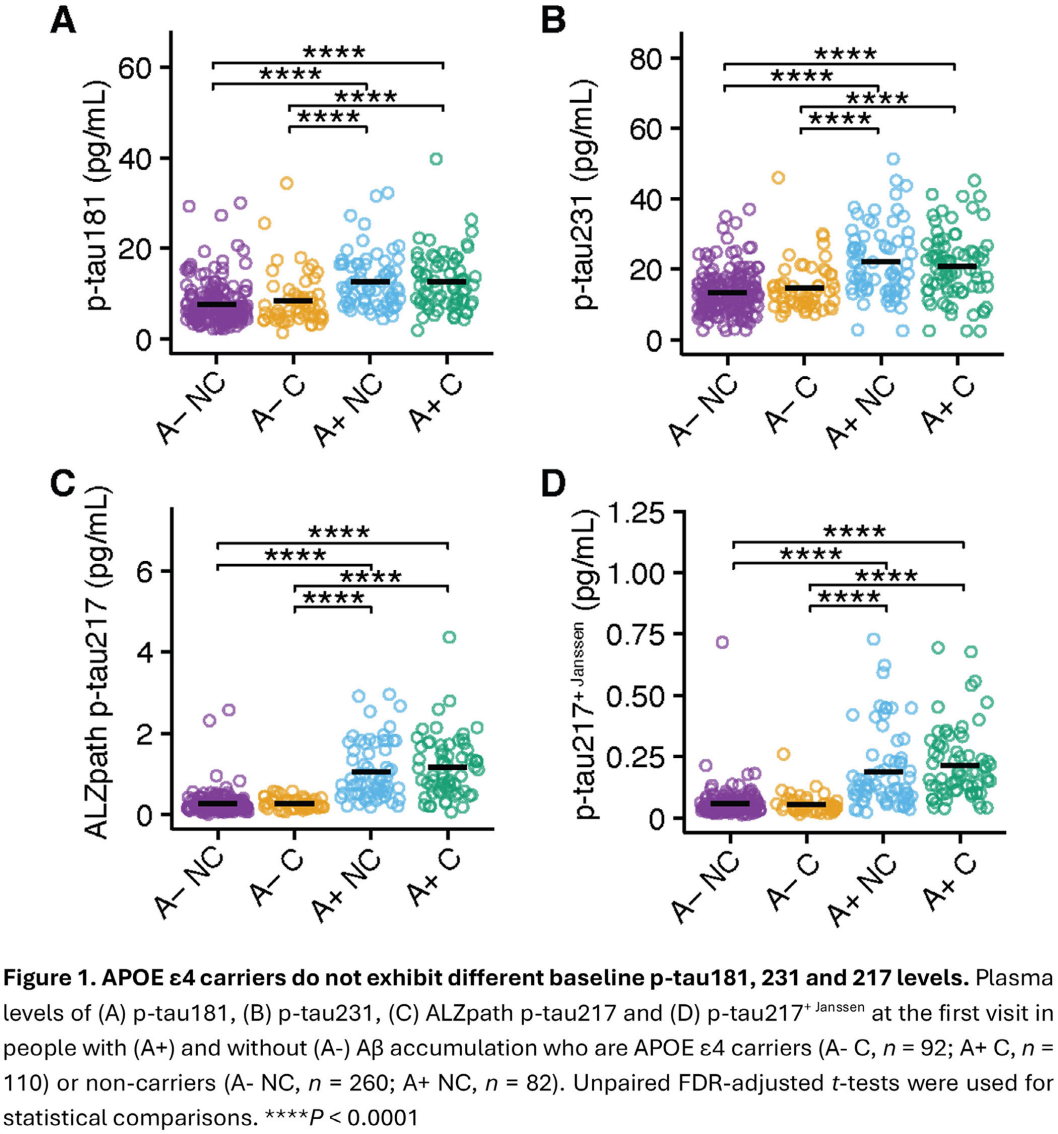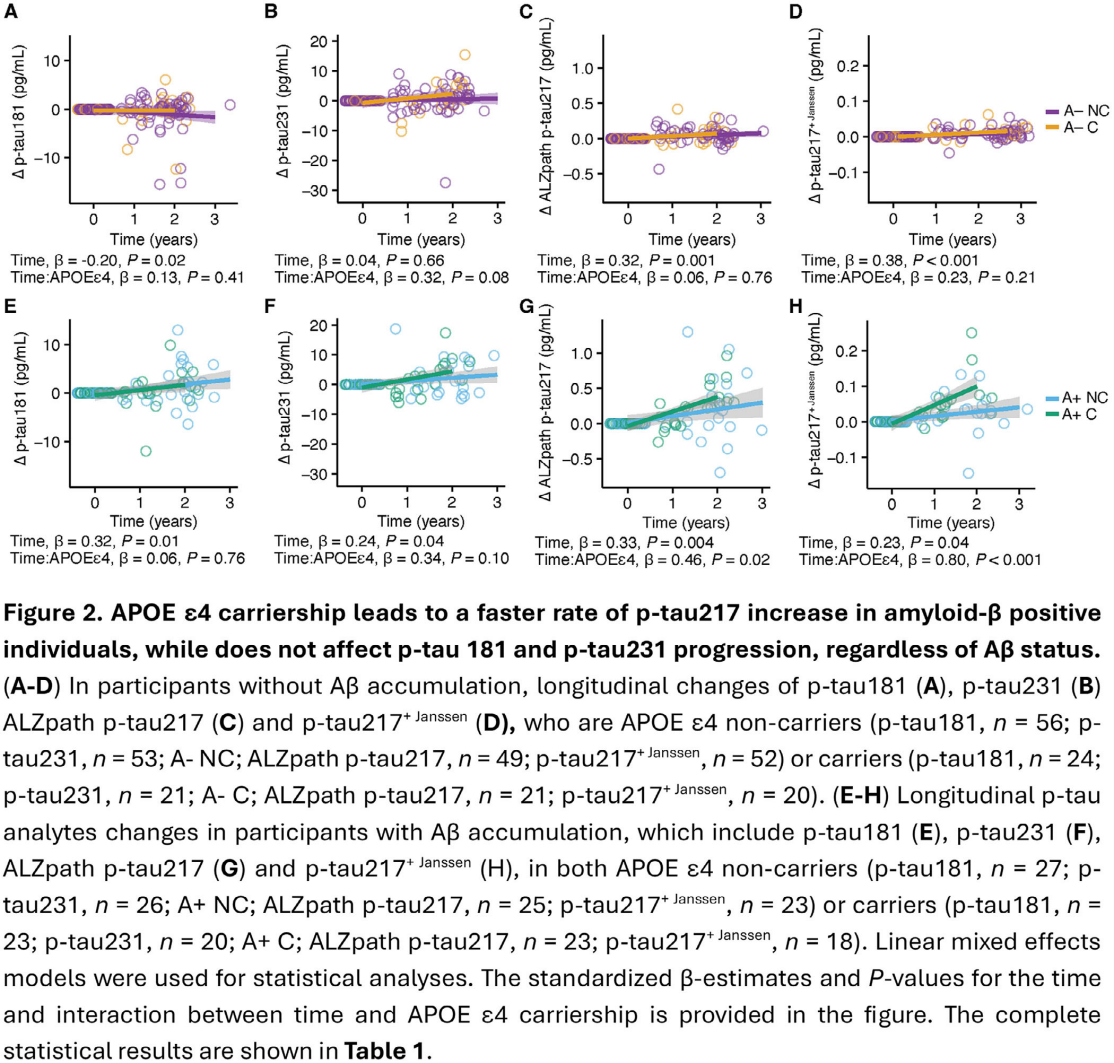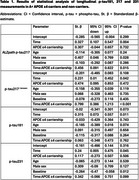# Accelerated rate of plasma *p*‐Tau217 progression in amyloid‐β APOE ɛ4 carriers

**DOI:** 10.1002/alz70856_102950

**Published:** 2025-12-25

**Authors:** Marina P Gonçalves, Tevy Chan, Arthur C. Macedo, Nesrine Rahmouni, Seyyed Ali Hosseini, Brandon J Hall, Yi‐Ting Wang, Etienne Aumont, Stijn Servaes, Cécile Tissot, Gleb Bezgin, Jaime Fernandez Arias, Lydia Trudel, Joseph Therriault, Kely Monica Quispialaya Socualaya, Jenna Stevenson, Yansheng Zheng, Firoza Z Lussier, Paolo Vitali, Gassan Massarweh, Jean‐Paul Soucy, Andrea Benedet, Henrik Zetterberg, Tharick A Pascoal, Marcel Woo, Pedro Rosa‐Neto, Translational Neuroimaging Laboratory

**Affiliations:** ^1^ Translational Neuroimaging Laboratory, The McGill University Research Centre for Studies in Aging, Montréal, QC, Canada; ^2^ Lawrence Berkeley National Laboratory, Berkeley, CA, USA; ^3^ Translational Neuroimaging Laboratory, The McGill University Research Centre for Studies in Aging, Montreal, QC, Canada; ^4^ The McGill University Research Centre for Studies in Aging, Montreal, QC, Canada; ^5^ McConnell Brain Imaging Centre ‐ McGill University, Montréal, QC, Canada; ^6^ McConnell Brain Imaging Centre, Montreal Neurological Institute and Hospital, McGill University, Montreal, QC, Canada; ^7^ Institute of Neuroscienace and Physiology, University of Gothenburg, Mölndal, Västra Götaland, Sweden; ^8^ Department of Psychiatry and Neurochemistry, Institute of Neuroscience and Physiology, The Sahlgrenska Academy at the University of Gothenburg, Mölndal, Västra Götalands län, Sweden; ^9^ University of Pittsburgh, Pittsburgh, PA, USA; ^10^ Department of Neurology, University Medical Centre Hamburg Eppendorf, Hamburg, Germany, Hamburg, Eppendorf, Hamburg‐Nord, Germany; ^11^ McGill University, Montreal, QC, Canada

## Abstract

**Background:**

The ɛ4 allele of the apolipoprotein E gene (APOE) increases susceptibility to Alzheimer's disease (AD) and is associated with reduced responsiveness to amyloid‐β (Aβ)‐removing therapies. In the context of AD diagnostics and disease‐modifying treatments, blood biomarkers have emerged as less invasive, accessible tools for monitoring disease status. This study tested the hypothesis whether APOEɛ4 carriers exhibit differences in plasma phosphorylated tau (*p*‐tau) levels compared to non‐carriers, considering the presence/absence of amyloidosis.

**Method:**

Participants enrolled in the Translational Biomarkers in Aging and Dementia (TRIAD) cohort, Montréal, Canada. Cerebral amyloid‐β and tau tangles were assessed using positron emission tomography (PET) imaging with [18F]AZD4694 and [18F]MK6240 tracers. Plasma *p*‐tau217 levels were quantified using commercially available assays (ALZpath and Janssen), while *p*‐tau181 and *p*‐tau231 were measured through in‐house Single Molecule Array (Simoa) method. Participants were classified as APOEɛ4 non‐carriers (NC) or carriers (C) and further stratified as amyloid‐positive (A+) or amyloid‐negative (A‐) based on PET cut‐offs (1.55) or CSF Aβ42/40 ratio (<0.068). Cross‐sectional analyses used unpaired FDR‐corrected t‐tests to compare baseline values between groups. Longitudinal analyses of biomarker progression employed mixed linear effects models.

**Result:**

554 participants were included in the cross‐sectional analysis (A‐NC=260; A‐C=92; A+NC=92; A+C=110). At baseline, no significant differences were observed between ɛ4 carriers and non‐carriers in plasma *p*‐tau181, *p*‐tau231, and *p*‐tau217 levels in either the A− or A+ groups (Figure 1). Longitudinal data (mean follow‐up: 26 months) from 224 participants (A‐NC=106; A‐C=35; A+NC=37; A+C=46) revealed no significant differences in progression rates for *p*‐tau181 and *p*‐tau231 between carriers and non‐carriers, regardless of amyloid status. However, both assays demonstrated accelerated increases in *p*‐tau217 levels among A+ participants (ALZpath: β=0.46, *p* = 0.02; Janssen: β=0.80, *p* <0.001), but not in A‐ individuals (Figure 2). Notably, we detected no significant association between *p*‐tau217 change and the interaction between amyloid beta and APOEɛ4.

**Conclusion:**

In the presence of amyloidosis, APOEɛ4 carriers accelerate the plasma *p*‐tau217 progression. In contrast, ɛ4 carriership in A+ individuals did not correlate with rates of *p*‐tau181 and *p*‐tau231. An additive interaction between APOEɛ4 carriership and Aβ status was observed. Further investigation is needed to understand the association between APOEɛ4 carriers and AD biomarkers progression.